# Psoas Abscess

**Published:** 1908-02-01

**Authors:** 


### Points in Surgery.

### PSOAS ABSCESS.

Cases occur from time to time in which the discovery of some definite sign of a psoas abscess is the first indication of spinal disease; and this in spite of the fact that the patient may have been under medical observation for some considerable time. It must be remembered that patients with tuberculous disease of the vertebral column do not always present the typical series of symptoms described in text-books in association with this disease. Children, for instance, often complain merely of vague pains in the back, of which no notice is taken by their parents or medical advisers, and schoolboys may only present in the early stages a disinclination or inability to join in the pastimes of their comrades, which is attributed to inherent laziness, and may be treated as such with lamentable consequences. In adults the vague symptoms of the early stages are often attributed to lumbago or to sciatica, or to that condition known as "muscular rheumatism," a phrase used by laity and, it is to be feared, by medical men alike to designate any pain in the muscular system, without reference to its causation or pathology. This is more than ever the case to-day when the greater part of the adult populace has its attention prominently attracted to the symptoms of these and allied conditions by the cunningly worded advertisements of proprietary medicines. The following case illustrates this point well. A man, aged fifty-five, began to notice pain in the lumbar region, which came on gradually, and in a few months became constant. He had read some of the advertisements referred to above, and he diagnosed his own case as one of muscular rheumatism. He treated himself with medicines and the application of liniments to his back. In the course of some months the pain involved the right leg, and he was then told that he had sciatica. Then he lost power in his legs, and was unable to walk. He was treated for this by a doctor and kept in bed for a month, after which he was so much improved that he was able to resume his occupation, that of a painter. The pain, however, never left him, and two years after the onset of his first symptom he sought medical advice for a painful swelling in the right lumbar region. When seen he had a fluctuating swelling occupying the situation of Petit's triangle on the right side. It was tender in the centre. In the left groin just below Poupart's ligament, and external to the femoral vessels, was a small fluctuating swelling. The fluctuation was communicated to a swelling just above Poupart's ligament. It was easily reducible, but returned at once as soon as the pressure was removed, even when he was in the recumbent position. There was no tenderness on percussion of the vertebral spines and no obvious rigidity, but the patient was unable to sit up in bed without supporting himself with his hands. Here, then, was a man with obvious tuberculous disease of the vertebral column and a double psoas abscess, whose symptoms had persisted for two years, and yet the nature of his trouble had never been suspected.

A psoas abscess is generally the result of direct infection of the muscle from disease of the lumbar vertebræ, from which it derives its origin, but this is not invariably the case. The disease may start in the dorsal region and track down, finally involving the psoas muscle by burrowing beneath the internal arcuate ligament, which is really the thickened upper edge of the anterior part of the sheath of the muscle. The fibres of the muscle are converted into a granuloma, whose cells ultimately caseate; thus an abscess is formed, whose wall consists of the sheath of the psoas muscle. As long as the tumour so formed is confined within the abdomen it is rarely diagnosed, except in patients already under observation for tuberculous disease of the spine. Sooner or later, however, it makes its way to the surface. There are numerous ways in which it is said to do this, but in actual practice it nearly always points in one of three places—(1) Just below Poupart's ligament external to the femoral vessels, (2) just above Poupart's ligament, or (3) in Petit's triangle. It will be seen, therefore, that its natural inclination is to become superficial retroperitoneally, and it does this at the places where the abdominal wall is weakest. Petit's triangle, for instance, is a weak spot in the posterior abdominal wall, where is a hiatus between the external oblique muscle and the latissimus dorsi. When it points in this situation the diagnosis is comparatively obvious, when the other signs of spinal disease are taken into consideration. Not so, however, when the abscess comes to the surface beneath Poupart's ligament. In this situation it may simulate several other surgical conditions; the only one of any real practical importance, however, is a femoral hernia. How can the two be distinguished? In both cases there is a soft swelling situated over the crural ring in the upper part of Scarpa's triangle; both are reducible and both give an impulse to the hand when the patient coughs. But there is one test which is absolutely reliable. If the swelling fluctuates, and that fluctuation can be obtained alternately with fluctuation in a mass above Poupart's ligament, it can be said with certainty that the case is one of psoas abscess. In the case quoted above the diagnosis was aided by the existence of a lumbar abscess on the right side, and although the simultaneous existence of a psoas abscess on both sides is not common, it would hardly have been reasonable to go out of one's way to look for an independent cause for the swelling on the left side. There are other distinctions, but none of them is so reliable. A hernia does not tend to recur at once after reduction; the swelling due to an abscess does, sometimes even, as in this case, when the patient is in the recumbent position. The swelling due to a psoas abscess is dull on percussion, whereas a hernia is resonant unless it contains mainly omentum; and, lastly, a hernia is said to return with a gurgle, but this only occurs if the coil contains gas, and in practice the sign is often found to be absent.

(*To be continued.*)

